# Mechanisms of Azole Resistance and Trailing in *Candida tropicalis* Bloodstream Isolates

**DOI:** 10.3390/jof7080612

**Published:** 2021-07-28

**Authors:** Pao-Yu Chen, Yu-Chung Chuang, Un-In Wu, Hsin-Yun Sun, Jann-Tay Wang, Wang-Huei Sheng, Yee-Chun Chen, Shan-Chwen Chang

**Affiliations:** 1 Department of Internal Medicine, National Taiwan University Hospital, Taipei 100, Taiwan; chenpaoyu@gmail.com (P.-Y.C.); weischuang@gmail.com (Y.-C.C.); uninwu@gmail.com (U.-I.W.); hysun@ntu.edu.tw (H.-Y.S.); wang.jt1968@gmail.com (J.-T.W.); whsheng@ntu.edu.tw (W.-H.S.); changsc@ntu.edu.tw (S.-C.C.); 2 Graduate Institute of Clinical Medicine, National Taiwan University College of Medicine, Taipei 100, Taiwan; 3 Department of Medicine, College of Medicine, National Taiwan University, Taipei 100, Taiwan; 4 National Health Research Institutes, Miaoli 350, Taiwan

**Keywords:** fluconazole, voriconazole, isavuconazole, multilocus sequence typing, ergosterol, efflux pump

## Abstract

Objectives: Azole-resistant *Candida tropicalis* has emerged in Asia in the context of its trailing nature, defined by residual growth above minimum inhibitory concentrations (MICs). However, limited investigations in *C. tropicalis* have focused on the difference of genotypes and molecular mechanisms between these two traits. Methods: Sixty-four non-duplicated *C. tropicalis* bloodstream isolates collected in 2017 were evaluated for azole MICs by the EUCAST E.def 7.3.1 method, diploid sequence type (DST) by multilocus sequencing typing, and sequences and expression levels of genes encoding *ERG11*, its transcription factor, *UPC2*, and efflux pumps (*CDR1*, *CDR2* and *MDR1*). Results: Isavuconazole showed the highest in vitro activity and trailing against *C. tropicalis*, followed by voriconazole and fluconazole (geometric mean [GM] MIC, 0.008, 0.090, 1.163 mg/L, respectively; trailing GM, 27.4%, 20.8% and 19.5%, respectively; both overall *p* < 0.001). Fourteen (21.9%) isolates were non-WT to fluconazole/voriconazole, 12 of which were non-WT to isavuconazole and clustered in clonal complex (CC) 3. Twenty-five (39.1%) isolates were high trailing WT, including all CC2 isolates (44.0%) (containing DST140 and DST98). All azole non-WT isolates carried the *ERG11* mutations A395T/W and/or C461T/Y, and most carried the *UPC2* mutation T503C/Y. These mutations were not identified in low and high trailing WT isolates. Azole non-WT and high trailing WT isolates exhibited the highest expression levels of *ERG11* and *MDR1*, 3.91- and 2.30-fold, respectively (both overall *p* < 0.01). Conclusions: Azole resistance and trailing are phenotypically and genotypically different in *C. tropicalis*. Interference with azole binding and *MDR1* up-regulation confer azole resistance and trailing, respectively.

## 1. Introduction

*Candida tropicalis* is among the four major *Candida* species responsible for candidaemia worldwide [1]. It is the most common aetiology of invasive candidiasis in patients with haematological malignancies. The proportion of *C. tropicalis* among *Candida* species causing candidaemia are relatively high in tropical Asia and Latin America compared to other continents [2,3]. Azole-resistant *C. tropicalis* clinical isolates have emerged worldwide [4,5,6,7,8,9,10,11]. This has become particularly problematic in the Asia-Pacific region since 2010 [4,5,7,10,11]. Clonal complex 3 (CC3) with high-level azole resistance were isolated from patients and environments [5,7,10,11].

Explorations into molecular mechanisms of azole resistance in *C. tropicalis* include mutations and/or up-regulation of *ERG11* gene encoding the azole target lanosterol 14-α-sterol-demethylase [6,9,12,13,14,15], gain-of-function mutations in *UPC2* gene which encodes a transcription factor of *ERG11* [8,16], and overexpression of genes encoding efflux pumps, including ATP binding cassette (ABC) transporters and major facilitator superfamily (MFS), including *MDR1* [13,14,15,16]. *C. tropicalis* has two ABC transporters (*CDR1* and *CDR2*) [17], while the role of *CDR2* in azole resistance is less characterized. These resistance mechanisms were also found in other *Candida* species, including *C. albicans* and *C. glabrata* [18,19].

Azole trailing is defined as reduced, but with persistent growth at azole concentrations above the minimum inhibitory concentrations (MICs) [20,21,22]. Specifically, *Candida* species display growth inhibition of 50–80% over a broad azole concentration range, defined as trailing growth [20,21,22], according to current European Committee on Antimicrobial Susceptibility Testing (EUCAST) and Clinical and Laboratory Standard Institute (CLSI) recommending that the MIC of azole against *Candida* species is determined after 24 h at the lowest drug concentration giving growth inhibition of ≥50% of that of the drug-free control. It is often difficult to distinguish between azole trailing and resistance, therefore, the European Committee on Antimicrobial Susceptibility Testing (EUCAST) broth microdilution (BMD) method recommended the spectrophotometric endpoint reading to minimize the impact of azole trailing on MIC determinations [23].

In addition to *C. albicans* and *C. glabrata*, *C. tropicalis* is one of *Candida* species commonly displaying azole trailing [20,21,24], while the degree of trailing among different azoles is seldom compared in *C. tropicalis*. Although azole trailing isolates appear to be azole susceptible by in vitro testing, the molecular mechanisms involving azole resistance have been observed in *C. albicans* trailing isolates [25]. However, evidence of whether the aforementioned resistance mechanisms also play roles in trailing is limited [22]. Clonality of *C. tropicalis* with azole trailing was less investigated and the results in the previous studies were contradictory [9,20].

This study was designed to determine the activity of different azoles and their degree of trailing for *C. tropicalis* bloodstream isolates using the EUCAST BMD method to ascertain their clonal relationships, and to compare the molecular mechanisms between azole resistance and trailing isolates.

## 2. Materials and Methods

### 2.1. Candida Tropicalis Isolates

We evaluated 64 non-duplicated *C. tropicalis* bloodstream isolates from patients with candidaemia admitted to the National Taiwan University Hospital in 2017 [7]. These isolates were identified by matrix-assisted laser desorption ionization–time of flight mass spectrometry (Bruker, Bremen, Germany) in the central laboratory. They were reconfirmed for this study using CHROMagar Candida (Becton, Dickinson and Company, Sparks, MD, USA), the Vitek 2 yeast identification systems (bioMérieux, Marcy-L’Etoile, France), and/or internal transcribed spacer (ITS) sequencing as previously described [7]. This study was approved by the Research Ethics Committees (NTUH-201502034RIND, 201805097RIND).

### 2.2. Antifungal Susceptibility Testing and Trailing

The MICs for fluconazole, voriconazole (Sigma, St. Louis, MO, USA) and isavuconazole (Toronto Research Chemicals, Toronto, ON, Canada) were determined according to the EUCAST E.Def 7.3.1 method [20] using Costar tissue culture-treated plates (catalog no. CLS3596; Corning Life Sciences, Wujiang, China). Ten dilution ranges of each azole were prepared according to ISO recommendations. *Candida parapsilosis* (ATCC 22019) and *Candida krusei* (ATCC 6258) were used as reference strains for quality control. The MICs of fluconazole and voriconazole for *C. tropicalis* were interpreted according to the EUCAST epidemiological cut-off values (ECOFFs) for wild-type (WT)/non-WT classification, 1 mg/L and 0.125 mg/L, respectively. The tentative ECOFF of isavuconazole in this report is 0.03 mg/L, which was categorized based on isavuconazole MICs > two dilutions above the MIC_50_ [26]. Azole trailing was calculated as the geometric mean of residual growth observed in the wells in which concentrations were above the MICs compared to the growth control (background OD subtracted) [21]. WT isolates were dichotomized as low and high trailing groups by the median percentage of trailing among them: 19.9%, 21.5%, and 28.5% for fluconazole, voriconazole, and isavuconazole, respectively.

### 2.3. Multilocus Sequence Typing

*C. tropicalis* genomic DNA was extracted and all isolates were typed using an MLST scheme described previously [7]. In brief, the sequences of the nucleotide primers were used for PCR amplification of six gene fragments. Combination of the results from the six gene fragments yielding unique DSTs were used to infer putative genetic relationships between *C. tropicalis* isolates using the goeBURST algorithm version 1.2.1 [27].

### 2.4. ERG11 and UPC2 Sequencing

*C. tropicalis* genomic DNA was used as the template for the amplification of the full-length coding region of the *ERG11* and *UPC2* genes. The primers for amplification and sequencing were designed as previously described with modifications (Supplementary Table S1) [28,29,30]. Nucleotide sequences of the amplicon were aligned with those of the *ERG11* gene of *C. tropicalis* ATCC 750 (GenBank accession number: M23673.1) and with those of the *UPC2* gene of *C. tropicalis* MYA-3404 (GenBank accession number: NW_003020056.1).

### 2.5. Quantitative RT-PCR

Total RNA was extracted from each isolate grown to the mid exponential phase (1 × 10^7^ cells/mL) in YPD medium using the Qiagen 74104 RNeasy Mini Kit (Qiagen Inc., Germantown, MD, USA) following the manufacturer’s instructions. Total RNA was quantified using Nanodrop 2000 (Thermo Fisher Scientific, Waltham, MA, USA). First-strand cDNA was synthesized from 50 ng of total RNA using a GoScript™ Reverse Transcription System (Promega A5000, Madison, WI, USA). Quantitative PCR of *ACT1*, *ERG11*, *UPC2*, *CDR1*, *CDR2*, and *MDR1* was performed in triplicate using the Power SYBR^®^ green PCR master (Thermo Fisher Scientific, Waltham, MA, USA) and an Applied Biosystems StepOnePlus (Applied Biosystems, Foster City, CA, USA). The primers used for quantitative RT-PCR were listed in Supplementary Table S1. Amplification conditions consist of 10 min of denaturation at 95 °C, followed by 40 cycles of 15 s at 95 °C and 60 s at 60 °C. The expression levels were normalized by *ACT1* expression. Relative gene expressions were calculated as the fold change of each isolate compared with the median expression levels of low trailing WT isolates.

### 2.6. Statistical Analysis

Geometric means of azole MICs, trailing and proportions of prior azole exposure within 30 days before onset of candidaemia were compared among low trailing WT, high trailing WT, and non-WT isolates using the one-way ANOVA with Bonferroni correction, while relative gene expressions among the three groups were compared with Kruskal–Wallis with Dunn’s multiple comparisons test. Correlations between azole MICs were calculated using Pearson correlation analyses after log2 transformation, and those between azole trailing and MDR1 expressions were calculated using the Spearman correlation analysis. All analyses were performed using Stata software (version 14; StataCorp, College Station, TX, USA). Two-sided *p* values less than 0.05 were considered significant.

## 3. Results

### 3.1. Azole Minimum Inhibitory Concentrations, Trailing, and Corresponding Genotypes

Among 64 *C. tropicalis* bloodstream isolates, isavuconazole was most active followed by voriconazole, and then fluconazole regarding MIC_90_ and geometric means of MICs (Table 1). Of 14 isolates (21.9%) classified as non-wild-type (WT) to fluconazole and voriconazole, 12 isolates were also classified as non-WT to isavuconazole. All azole non-WT isolates were categorized as resistant to fluconazole/voriconazole by EUCAST clinical breakpoints. The strongest correlation was observed between fluconazole MIC and voriconazole MIC (R^2^ of 0.967), followed by those between isavuconazole and voriconazole/fluconazole (R^2^ of 0.766 and 0.719, respectively; All *p* < 0.001).

**Figure 1:**
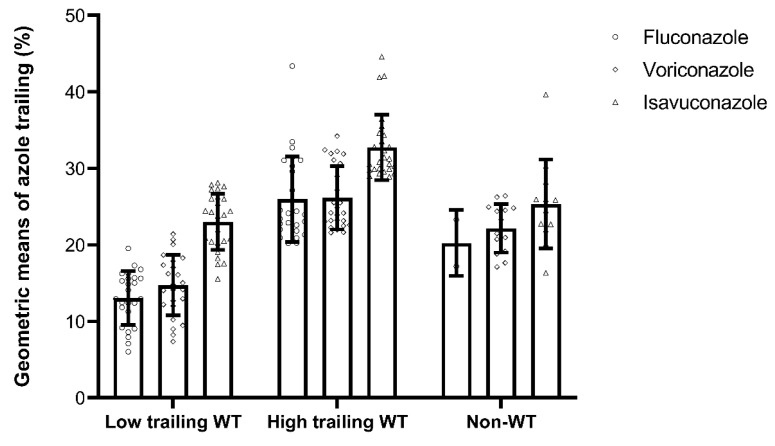
Geometric means of trailing percentages for fluconazole, voriconazole, and isavuconazole among low and high trailing WT and non-WT *C. tropicalis* isolates. Isolates with fluconazole MICs of ≥64 m/L were excluded from calculating the percentage of trailing due to out-of-dilution ranges. Error bars indicate standard deviations.

Overall, isavuconazole displayed the highest mean percentages of trailing, followed by voriconazole and fluconazole (Table 1), and the orders of trailing degree among azoles remained similar in low and high trailing WT and non-WT subgroups (Figure 1). Additionally, the degree of trailing for each azole was highest in high trailing WT isolates compared to those in non-WT and low trailing WT isolates (Figure 1), suggesting high trailing was not misclassified as non-WT.

As shown in Table 2 and Supplementary Figure S1, azole non-WT isolates were mainly within CC3, none of which displayed trailing growth (Supplementary Figure S2). This suggested fluconazole non-WT in this clone was not attributed to heavy trailing growth of >50%, also called resistant heavily trailing, over a broad fluconazole concentration range [22]. On the other hand, all 11 CC2 isolates were classified as high trailing WT to three azoles, except one as a low trailing WT to isavuconazole, and contributed 44.0% of high trailing to fluconazole/voriconazole WT isolates. Of note, fluconazole/voriconazole non-WT isolates had a greatest proportion of prior azole exposure within 30 days before candidaemia onset, followed by those in high and low trailing WT isolates (57.1% vs. 28.0% vs. 4.0%, overall *p* < 0.001).

### 3.2. ERG11 and UPC2 Sequencing

Twelve azole non-WT CC3 isolates possessed homozygote and/or heterozygote nonsynonymous mutations, A395T/W and C461T/Y (Table 3), corresponding to Y132F and S154F substitutions, respectively. Only one nonsynonymous mutation in the *UPC2* gene, T503Y/C, was exclusively detected in azole non-WT CC3 isolates. These isolates displayed high fluconazole and voriconazole MICs of ≥64 mg/L and of 4 mg/L, respectively, and non-WT to isavuconazole. The remaining two azole non-WT non-CC3 isolates sharing A395T/W and/or C461T/Y displayed relatively low fluconazole and voriconazole MICs (8–16 mg/L, and 0.5–1 mg/L, respectively), and WT to isavuconazole. All low and high trailing WT isolates did not have the above *ERG11* and *UPC2* mutations.

### 3.3. Expression of ERG11, UPC2, and Genes Encoding Efflux Pumps

**Figure 2:**
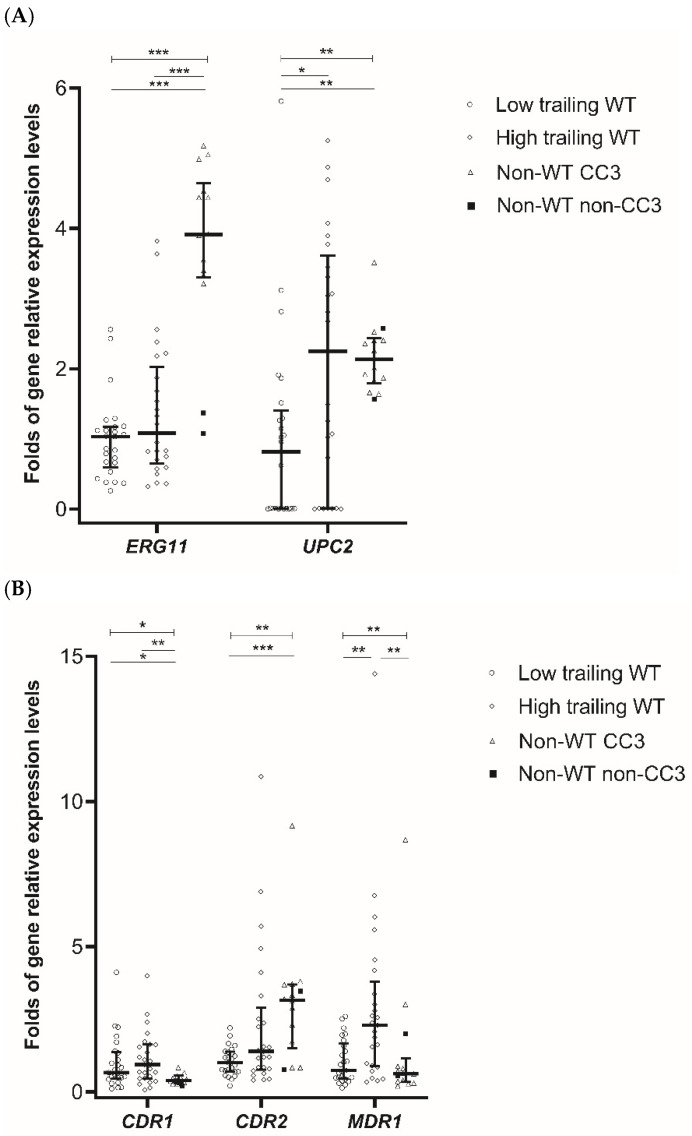
Expression levels of genes encoding the azole target or its transcription factor, *ERG11* and *UPC2* (**A**), and genes encoding efflux pumps, *CDR1*, *CDR2*, and *MDR1* (**B**) of low and high trailing WT, and non-WT *C. tropicalis* isolates. Horizontal lines and error bars indicate medians and interquartile, respectively. Asterisks indicate statistical significance (*: *p* < 0.05; **: *p* < 0.01; ***: *p* < 0.001).

Gene expression levels of *ERG11* and *UPC2* differed significantly among azole non-WT isolates and low and high trailing WT isolates (both overall *p* < 0.05) (Figure 2A). Azole non-WT isolates exhibited 3.91-fold higher median expression levels of the *ERG11* gene than those of low and high trailing WT isolates (both *p* < 0.001) (Figure 2A).

Azole non-WT isolates exhibited the highest and lowest median expression levels of the *CDR2* and *CDR1* genes compared to low and high trailing WT isolates, while there was no difference between those in low and high trailing WT isolates (Figure 2B). On the other hand, high trailing WT isolates exhibited the highest median expression levels (2.30-fold) of *MDR1* than those of low trailing WT and non-WT isolates (overall and pair-wised *p* < 0.05). Furthermore, the degree of trailing was positively correlated with *MDR1* expression levels (r  =  0.346; *p*  =  0.005) (Supplementary Figure S3).

Among 14 azole non-WT isolates, CC3 isolates demonstrated higher expression levels of *ERG11* but similar expression levels of other genes compared to those of non-CC3 isolates (Figure 2A,B).

## 4. Discussion

This study confirmed previous studies that isavuconazole was most potent against *C. tropicalis* among three azoles evaluated while its non-WT showed cross-resistance to fluconazole and voriconazole [26,31]. Isavuconazole displayed the highest degree of trailing among different azoles against *C. tropicalis* [20,21]. This study distinguished azole non-WT from high trailing WT and demonstrated that genotypes and molecular mechanisms varied between them. Azole non-WT isolates had nonsynonymous mutations and/or overexpression of the *ERG11* gene, alongside with up-regulations of *CDR2*, while up-regulation of *MDR1* was mainly found in high trailing WT isolates.

Overall, in vitro activities for three azoles tested against *C. tropicalis* in this study were consistent with previously published EUCAST MICs [21,26,31]. Given isavuconazole shares more similar chemical structures with short-chained azoles, our and other reports [26] demonstrated good correlation between isavuconazole and fluconazole/voriconazole. This is of particular concern in view of the emerging pan-azole-resistant clone, CC3, in Asia [5,7,10,11]. As shown in this study, *C. tropicalis* isolates exhibiting cross-resistance between isavuconazole and other azoles were collected in 2017, while isavuconazole was introduced in Asia in 2019. As antifungal susceptibility testing is not routinely performed in many Asian hospitals [32] and azoles are step-down oral agents for invasive candidiasis, clinicians should be aware of the possibility of pan-azole resistance in *C. tropicalis*.

Azole trailing in *Candida* is a common phenomenon which has been evaluated by various methodologies and reading endpoints (24 vs. 48 h) [9,20,21,22,24,25,33,34]. Our results were consistent with previous reports that quantification of trailing among different *Candida* strains or antifungal agents was feasible by performing the 24 h EUCAST BMD method [20,21,22]. Marcos-Zambrano L.J. et al. showed *C. tropicalis* displayed a higher degree of trailing to fluconazole and isavuconazole among different *Candida* species [20,21]. We further demonstrated that *C. tropicalis* produced the highest degree of trailing to isavuconazole among various azoles, and those to fluconazole and voriconazole were comparable.

CC2 was most prevalent among high trailing WT isolates in a diverse genetic background (Table 2 and Supplementary Figure S1). DST140 fluconazole WT isolate was first identified in 1987 at this hospital (data not shown). Later, another Taiwan surveillance study in 1999 demonstrated fluconazole trailing growth clustered in the CC2 clone, mainly containing DST98 and DST140 [34]. Additionally, our previous study has demonstrated CC2 as the second largest clone after CC3 without expansion by nosocomial transmission from 2011 to 2017 in Taiwan [7]. Whether this trailing nature is genotype-specific warrants further exploration, but our findings and others [9,20,34] suggest that geographic variation in *C. tropicalis* genotypes should be considered for variations in trailing growth from different studies.

As for molecular mechanisms of azole resistance, this study found that all 14 azole non-WT isolates had the specific *ERG11* mutations, which supports the current EUCAST fluconazole/voriconazole ECOFFs for *C. tropicalis*. These *ERG11* mutations resulting in Y132F and/or S154F substitutions were concordant to previous studies [9,12,13,14,15]. Y132F alternation is located on the exposed active-site cavity of the Erg11 protein. This results in interference with azole binding and subsequent azole resistance across different *Candida* spp., including multi-drug resistant *Candida auris* and *C. albicans* [17,18,19,35]. A *Saccharomyces cerevisiae* model also has confirmed that Y132F can cause azole resistance in *C. tropicalis* [12,14].

In this report, wide variations of fluconazole MICs among azole non-WT isolates may indicate that *ERG11* mutations *per se* do not fully account for azole resistance levels. In this study, coexistence of *ERG11* overexpression and *ERG11* mutations in CC3 conferred high-level resistance to fluconazole/voriconazole and non-WT to isavuconazole, while the other two azole non-WT non-CC3 isolates in the absence of *ERG11* overexpression showed moderate resistance to fluconazole/voriconazole and WT to isavuconazole. Given we found only CC3 isolates carried the *UPC2* mutation T503C/Y and *ERG11* overexpression and other studies also showed specific *UPC2* mutations in azole-resistant *C. tropicalis* [8,16], we speculated the gain-of-function mutations of *UPC2* may play roles in regulating *ERG11* expression.

Overexpression of different efflux pumps, either *CDR1* or *MDR1*, in azole-resistant *C. tropicalis* has been reported previously [13,15,16,22], while our study was first to demonstrate up-regulations of *CDR2* and *MDR1* expression in azole non-WT and high trailing WT isolates, respectively. This is concordant to a *S. cerevisiae* model showing ABC transporters, but not *MDR1* conferring azole resistance in *C. albicans* and *C. glabrata* [35]. Additionally, a previous study demonstrated up-regulation of ABC transporters not related to the degree of trailing in *C. tropicalis* [22]. Our finding, showing the positive correlation between the degree of trailing and *MDR1* expression levels in *C. tropicalis*, also supported the role of MFS up-regulation in azole trailing.

This finding further suggests trailing is a unique trait depending on azole induction. This in conjunction with the *Galleria* model showing reduced fluconazole activities against both moderate and heavy azole trailing *C. tropicalis* isolates (defined by trailing of 26–50% and >50%, respectively) [22] raised the concern about the efficacy of azole in treatment for azole trailing *C. tropicalis* infections. Although the *Galleria* model did not show a significant survival difference between voriconazole trailing and susceptible *C. albicans* strains as treated by voriconazole [36], clinical studies have shown azole tolerant *C. albicans* cause persistent candidaemia or higher mortalities [33,37]. However, clinical outcomes of candidaemia patients infected by azole trailing *C. tropicalis* were not different, even better, compared to those infected by azole non-trailing isolates in relatively small cohorts [9,38]. Hence, this is warranted to determine the impact of azole trailing strains on patients with *C. tropicalis* bloodstream infections to guide treatment choices.

Our study had some limitations. First, our observations were generated mainly based on a unique clonal complex of azole non-WT and high trailing WT. These findings are likely unable to be generalized to other countries in the absence of the characterization of genotypes of *C. tropicalis*. Second, heavily trailing (>50%) *C. tropicalis* not identified in this study also limits generalization of our results. Also, the threshold value to define high and low trailing WT is specific for the collection of isolates used in this study, it may not be applicable to other laboratories. Additionally, our findings may not be generalized to *C. albicans* given other studies have shown both ABC transports and MFS up-regulation confer azole resistance in *C. albicans* [18,19,39]. We examined the potential mechanisms of azole resistance and trailing in *C. tropicalis* as thoroughly as possible, but molecular mechanisms of these two distinct traits are more complicated. For example, genomic structure variations, either aneuploidy or copy number variations of drug resistant genes, also confer azole resistance and/or trailing [17,40]. Notably, mutations of transcription factors regulating efflux pumps, including *TAC1* and *MRR1*, have been identified in azole resistance *C. tropicalis* [9], while their roles in azole trailing have yet to be defined. Furthermore, efflux pump activities in azole trailing may only reflect the downstream phenomenon of variable stress responses as *Candida* species expose to azoles [33].

Collectively, our results supported not only EUCAST fluconazole/voriconazole ECOFFs for *C. tropicalis*, but also the EUCAST BMD method to quantify the degree of azole trailing. Furthermore, our results underscored azole resistance and trailing of *C. tropicalis* are phenotypically and genotypically different as shown in *C. albicans*. Most azole non-WT isolates belong to CC3, while CC2 is the most prevalent clone among high trailing WT isolates. Interference with azole binding and *MDR1* up-regulation were the major mode of resistance and the degree of trailing, respectively. Deep insights to underlying mechanisms and clinical significance of azole resistance and trailing in *C. tropicalis* bloodstream isolates are worth exploring further.

## Figures and Tables

**Table 1:** Minimum inhibitory concentrations (mg/L), proportions of non-wild-type, and percentage of trailing for different azoles against 64 *Candida tropicalis* bloodstream isolates.

	Fluconazole (FLC)	Voriconazole (VRC)	Isavuconazole (ISA)			*p*-Value	
				Overall	FLC vs. VRC	FLC vs. ISA	VRC vs. ISA
**MIC Range ^a,b^**	≤0.125–>64	≤0.015–4	≤0.002–0.125				
**MIC_50_/MIC_90_**	0.5/>64	0.06/4	0.008/0.125				
**GM MIC**	1.163	0.090	0.008	<0.001	<0.001	<0.001	<0.001
**% of Non-WT ^c^ Isolates(No. of Isolates)**	21.9 (14)	21.9 (14)	18.8 (12)				
**GM (±SD) of Trailing (%)**	19.5 (±7.9) ^d^	20.8 (±6.4)	27.4 (±6.3)	<0.001	>0.99	<0.001	<0.001

Abbreviations: MIC_50_/MIC_90_, MIC for 50% and 90% of the respective population; GM, geometric mean; non-WT, non-wild-type; SD, standard deviation. ^a^ The 10-dilution range of each azole are listed as follows: fluconazole, ≤0.125 mg/L–64 mg/L; voriconazole, ≤0.015 mg/L–8 mg/L; isavuconazole, ≤0.002 mg/L–1 mg/L. ^b^ Quality control strains were tested two times, and results for each azole against each quality control strain are as follows: *C. krusei* ATCC 6258, fluconazole, 32 mg/L (twice) (target [range] 32 [16–64] mg/L); voriconazole 0.25 mg/L (twice) (0.06–0.125 [0.03–0.25] mg/L); isavuconazole, 0.03 and 0.06 mg/L (once for each) (0.03 [0.016–0.06] mg/L); *C. parapsilosis* ATCC 22019, fluconazole, 0.5 and 1 mg/L (once for each) (1 [0.5–2] mg/L); voriconazole 0.03 mg/L (twice) (0.03 [0.016–0.06] mg/L); isavuconazole, 0.008 mg/L (twice) (0.016 [0.008–0.03] mg/L). ^c^ The current EUCAST epidemiological cut-off values (ECOFFs) of fluconazole and voriconazole for *C. tropicalis* are 1 mg/L and 0.125 mg/L. The tentative ECOFF of isavuconazole in this report is 0.03 mg/L. ^d^ Isolates with fluconazole MICs ≥ 64 m/L were excluded from the calculating percentage of trailing due to out-of-dilution ranges.

**Table 2:** Distributions of low and high trailing wild-type, and non-wild-type *Candida tropicalis* isolates, and their corresponding clonal complexes ^a^ and minimum inhibitory concentrations (mg/L).

	Fluconazole			Voriconazole			Isavuconazole		
	Low Trailing WT	High Trailing WT	Non-WT	Low Trailing WT	High Trailing WT	Non-WT	Low Trailing WT	High Trailing WT	Non-WT
**Clonal complex ^b^ (No. of Isolates)**									
1 (4)	4	0	0	4	0	0	4	0	0
2 (11)	0	**11**	0	0	**11**	0	1	**10**	0
3 (14)	2	0	12	0	2	12	0	2	12
4 (10)	5	5	0	6	4	0	4	6	0
6 (2)	1	1	0	2	0	0	1	1	0
8 (2)	0	2	0	0	2	0	0	2	0
9 (2)	2	0	0	2	0	0	2	0	0
11 (1)	0	0	1	0	0	1	1	0	0
20 (2)	2	0	0	1	1	0	2	0	0
22 (3)	2	1	0	2	1	0	1	2	0
28 (1)	1	0	0	1	0	0	1	0	0
35 (1)	1	0	0	1	0	0	1	0	0
41 (1)	0	0	1	0	0	1	0	1	0
49 (1)	1	0	0	1	0	0	1	0	0
63 (1)	0	1	0	0	1	0	1	0	0
Singletons (8)	4	4	0	5	3	0	6	2	0
Total (64)	25	25	14	25	25	14	26	26	12
**MIC ^c^**									
GM	0.330	0.379	>64	0.269	0.418	3.123	0.004	0.006	0.104
MIC_50_	0.25	0.5	>64	0.03	0.06	4	0.006	0.008	0.125
MIC_90_	0.5	0.5	>64	0.06	0.06	4	0.015	0.015	0.125
Range	≤0.125–0.5	≤0.125–0.5	8– >64	≤0.015–0.06	≤0.015–0.06	0.5–4	≤0.002–0.015	≤0.002–0.015	0.06–0.125

Abbreviations: DST, diploid sequence type; non-WT, non-wild-type; MIC, minimum inhibitory concentration; GM, geometric men; MIC_50_/MIC_90_, MIC for 50% and 90% of the; MIC, minimum inhibitory concentration; GM, geometric men; MIC_50_/MIC_90_, MIC for 50% and 90% of the respective population. ^a^ The most prevalent wild-type clone with high trailing is highlighted in boldface type, and the most dominant non-wild-type (WT) clone is highlighted with gray shading. ^b^ Clonal complex is determined by multilocus sequence typing when five of the six alleles are identical between a pair as described previously [7]. In this study, CC2 included diploid sequence type (DST)98 (*n* = 3), DST140 (*n* = 5), DST820 (*n* = 1), DTS829 (*n* = 1), and DST830 (*n* = 1). Twelve of 14 isolates in CC3 classified as non-WT to three tested azoles included DST376, DST505, DST507, and DST838. The other two in CC3 categorized as WT to three tested azoles included DST333 and DST847 as described previously [7]. ^c^ Quality control strains were tested 2 times, and results for each azole against each quality control strain were as followed: *C. krusei* ATCC 6258, fluconazole, 32 mg/L (twice) (target [range] 32 [16–64] mg/L); voriconazole 0.25 mg/L (twice) (0.06–0.125 [0.03–0.25] mg/L); isavuconazole, 0.03 and 0.06 mg/L (once for each) (0.03 [0.016–0.06] mg/L); *C. parapsilosis* ATCC 22019, fluconazole, 0.5 and 1 mg/L (once for each) (1 [0.5–2] mg/L); voriconazole 0.03 mg/L (twice) (0.03 [0.016–0.06] mg/L); isavuconazole, 0.008 (twice) (0.016 [0.008–0.03] mg/L).

**Table 3:** Homozygote and heterozygote nonsynonymous mutations in *ERG11* gene and *UPC2* gene among 14 *ERG11* non-wild-type *Candida tropicalis* isolates with azole MICs and corresponding clonal complexes.

	*ERG11* Gene ^a^		*UPC2* Gene ^a^	Clonal Complex (No. of Isolates)	MIC Range (mg/L) ^d^		
Nucleotide Position	395	461	503		FLC	VRC	ISA
	A	C	**T**	Reference ^b^			
	**W**	**Y**	**Y**	CC3 ^c^ (11)	>64	4	0.06–0.125
	**W**	**Y**	**C**	CC3 ^c^ (1)	64	4	0.125
	**T**	**T**	T	CC41 ^c^ (1)	16	1	0.015
	A	**T**	T	CC11 ^c^ (1)	8	0.5	0.008

Abbreviations: MIC, minimum inhibitory concentrations; FLC, fluconazole; VRC, voriconazole; ISA, isavuconazole; CC, clonal complex. ^a^ Nonsynonymous mutations are highlighted in boldface. Heterozygotes (K, M, R, S, W, and Y) are defined by the International Union of Pure and Applied Chemistry (https://iupac.org; accessed on 30 August 2020) nomenclature. The meaning of R, W, and Y refer to AG, AT, and CT, respectively. ^b^ Reference strain sequence: *ERG11* gene: ATCC750, GeneBank accession no.: M23673.1; *UPC2* gene: MYA3404, GenBank accession no.: NW_003020056.1. ^c^ Clonal complex is determined by multilocus sequence typing when five of the six alleles are identical between a pair as described previously [7]. In this study, 12 isolates in CC3 were classified as non-wild-type (WT) to three tested azoles, including the diploid sequence type (DST) 376, DST505, DST507, and DST838. Two isolates classified as non-WT to fluconazole and voriconazole, but WT to isavuconazole, belonged to DST508 in CC11, and DST819 in CC41, respectively. ^d^ Quality control strains were tested 2 times, and results for each azole against each quality control strain were as followed: *C. krusei* ATCC 6258, fluconazole, 32 mg/L (twice) (target [range] 32 [16–64] mg/L); voriconazole 0.25 mg/L (twice) (0.06–0.125 [0.03–0.25] mg/L); isavuconazole, 0.03 and 0.06 mg/L (once for each) (0.03 [0.016–0.06] mg/L); *C. parapsilosis* ATCC 22019, fluconazole, 0.5 and 1 mg/L (once for each) (1 [0.5–2] mg/L); voriconazole 0.03 mg/L (twice) (0.03 [0.016–0.06] mg/L); isavuconazole, 0.008 (twice) (0.016 [0.008–0.03] mg/L).

## Data Availability

All data are available within the article and Supplementary Materials.

## Supplementary Materials

The followings are available online at https://www.mdpi.com/2309-608X/7/8/612/s1, Figure S1: Minimum spanning tree of 64 *C. tropicalis* bloodstream isolates, Figure S2: Overview of the EUCAST microdilution ELISA read-outs of the twelve isolates in clonal complex 3, Figure S3: Scatter plot between geometric means of trailing percentages and *MDR1* expression levels, Table S1: Primers for sequencing and quantitative RT-PCR.
