# Correction: Chen et al. Mechanisms of Azole Resistance and Trailing in *Candida tropicalis* Bloodstream Isolates. *J. Fungi* 2021, *7*, 612

**DOI:** 10.3390/jof7110932

**Published:** 2021-11-02

**Authors:** Pao-Yu Chen, Yu-Chung Chuang, Un-In Wu, Hsin-Yun Sun, Jann-Tay Wang, Wang-Huei Sheng, Yee-Chun Chen, Shan-Chwen Chang

**Affiliations:** 1 Department of Internal Medicine, National Taiwan University Hospital, Taipei 100, Taiwan; chenpaoyu@gmail.com (P.-Y.C.); weischuang@gmail.com (Y.-C.C.); uninwu@gmail.com (U.-I.W.); hysun@ntu.edu.tw (H.-Y.S.); wang.jt1968@gmail.com (J.-T.W.); whsheng@ntu.edu.tw (W.-H.S.); changsc@ntu.edu.tw (S.-C.C.); 2 Graduate Institute of Clinical Medicine, National Taiwan University College of Medicine, Taipei 100, Taiwan; 3 Department of Medicine, College of Medicine, National Taiwan University, Taipei 100, Taiwan; 4 National Health Research Institutes, Miaoli 350, Taiwan

Because the geometric means of fluconazole and voriconazole in some isolates were not correctly input into the low trailing or high trailing WT group during the visualization process when using GraphPad Prism (version 9.2.0; GraphPad Software, San Diego, CA, USA), the authors wish to make the following corrections to this paper [1], which was published in *Journal of Fungi*. Figure 1 should be replaced with the version below. The addition of this correction does not change any of the statistics reported in the original analysis that were determined using Stata software (version 14; StataCorp, College Station, TX, USA).

All co-authors agree with the content of this correction and wish to apologize for any inconvenience to the readers resulting from this error.

## Figures and Tables

**Figure 1:**
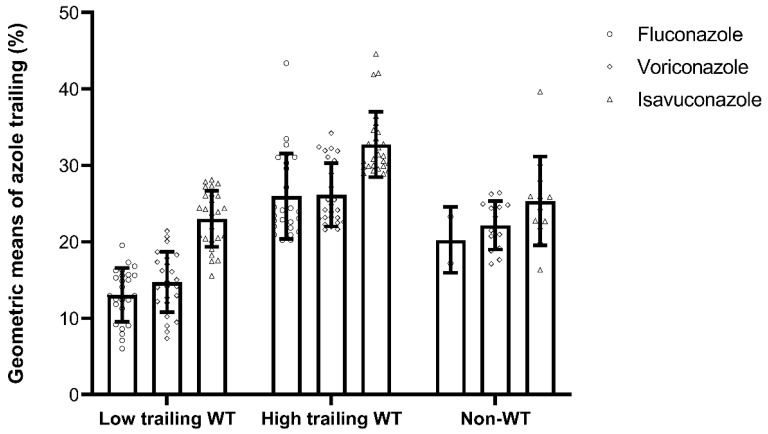
Geometric means of trailing percentages for fluconazole, voriconazole, and isavuconazole among low and high trailing WT and non-WT *C. tropicalis* isolates. Isolates with fluconazole MICs of ≥64 m/L were excluded from calculating the percentage of trailing due to out-of-dilution ranges. Error bars indicate standard deviations.
