# Supplementary material for: Mechanisms of Azole Resistance and Trailing in Candida tropicalis Bloodstream Isolates
Source: J Fungi (Basel). 2021 Jul 28;7(8):612. doi: 10.3390/jof7080612 (PMC8396981; doi:10.3390/jof7080612)
Supplement: Supplementary file 1 [file jof-07-00612-s001.zip › Supplementary.pdf]

Mechanisms of Azole Resistance and Trailing in *Candida tropicalis* Bloodstream Isolates

Supplementary

Supplementary Table···2

- Supplementary Table. Primers for sequencing and quantitative RT-PCR

Supplementary Figure···4

- Supplementary Figure S1. Minimum spanning tree of 64 *C. tropicalis* bloodstream isolates.
- Supplementary Figure S2. Overview of the EUCAST microdilution ELISA read-outs of the twelve isolates in clonal complex 3.
- Supplementary Figure S3. Scatter plot between geometric means of trailing percentages and *MDR1* expression levels.

References···7

Supplementary Table S1. Primers used in this study

Gene name	GenBank accession no.	Application	Primer	Nucleotide sequence (5' to 3')	Nucleotide coordinates	Reference
Ct*ERG11*[a]	M23673.1	Amplification & Sequencing of *ERG11* gene	ERG11-2F	ATCCCACAGGCTTATTTGAAA	+521 to +541	[1]
			ERG11-2R	GGTCTCTTTCCTTGGTTTTG	+1170 to +1189	[1]
			ERG11-3F	TGCTGAAGAAGCTTATACCC	+981 to +1000	[1]
			ERG11-3R	CAAGGAATCAATCAAATCTCTC	+1461 to +1482	[1]
			ERG11-4F	GGTGGTCAACATACTTCTGC	+1561 to +1580	[1]
			ERG11-4R	AGCAGGTTCTAATGGTAAGG	+2171 to +2190	[1]
			ERG11-5F	AAACGGTGATAAGGTTCCAG	+2127 to +2146	[1]
			ERG11-5Rb	TGCGGTACAGGTGATCTGTG	+2445 to + 2464	[2]
Ct*ERG11*	XM_002550939.1	Quantitative RT-PCR of *ERG11* gene	ERG11-F	GATAAAGGTTTCACCCCAATCAACT	+673 to +697	This study
			ERG11-R	CCTTCATGTAATGAGCAGATATCTTTCT	+751 to +778	This study
Ct*UPC2*	NW_003020056.1	Amplification & Sequencing of *UPC2* gene	UPC2-F1	CCACTACCATCACTATCATCAC	+308640 to +308661	[3]
			UPC2-R3	CAATACCTATTGTGATTACTTC	+306106 to +306127	[3]
			UPC2-F5	TCAGAAGCTAATGGCCCA	+308224 to +308241	[3]
			UPC2-R5	GGATATTCATCTTGGCAGC	+308082 to +308103	[3]
			UPC2-F4	ACCCCTACATCCATTCCA	+307804 to +307821	[3]
			UPC2-R6	CACGCAATAATCGCAAGG-	+307032 to +307049	[3]
			UPC2-R3	CAATACCTATTGTGATTACTTC	+306106 to +306127	[3]
Ct*UPC2*	XM_002548816.1	Quantitative RT-PCR of *UCP2* gene	UPC2-F	GAGTGGAACAACAACACAACAA	+369 to +390	[4]
			UPC2-R	TAAATCCCCTAAACCTGAAAGA	+555 to +576	[4]
Ct*CDR1*	XM_002548263.1	Quantitative RT-PCR of *CDR1* gene	CDR1-F	AAAATACGGAGCTGATCCATGTC	+3288 to +3310	This study
			CDR1-R	CGGGTGATTTCATCGTGAACT	+3423 to +3443	This study
Ct*CDR2*	XM_002547994.1	Quantitative RT-PCR of *CDR2* gene	CDR2-F	CCAATTGCAACCATTTTATCACA	+1534 to +1556	This study

			CDR2-R	AAAGTGAACCAGATCTGAAATAGAATGTA	+1614 to + 1642	This study
Ct*MDR1*	XM_002548069.1	Quantitative RT-PCR of *MDR1* gene	MDR1-F	GGAGATGGACTTTTTGGTTTATGTGTAT	+776 to +803	This study
			MDR1-R	CGACGCAATAAAGTCTTACTGAAAGA	+850 to +875	This study
Ct*ACT*	XM_002549283.1	Quantitative RT-PCR of *ACT* gene	ACT-F	TGATGGTGTTACCCACGTTGTC	+486 to +507	This study
			ACT-R	TCATCAAGTAGTCGGTCAAGTCTCTAC	+563 to +589	This study

[a] Primers of *ERG11* gene are designed to cover coding sequence (nucleotide coordinates +643—+2229) from *C. tropicalis* (GenBank accession no.: M23673.1).

Supplementary Figure S1.

Minimum spanning tree of 64 *C. tropicalis* bloodstream isolates. Each circle corresponds to a diploid sequence type (DST), and DST numbers are depicted nearby circles. The size of the circle indicates the number of the isolates belonging to a specific DST and classified as azole non-wild type (WT) (red), high trailing WT (yellow), or low trailing WT (white). Lines between circles indicate the similarity between profiles: bold lines indicate 5 of 6 alleles are identical, solid lines indicate 4 alleles are identical, dashes lines indicate 3 alleles are identical, and dotted lines indicate ≤2 alleles are similar. Shaded areas indicate groups of target clonal complexes (CCs): green shading indicate CC3, purple shading indicate CC2, and pink shading indicate CC4.

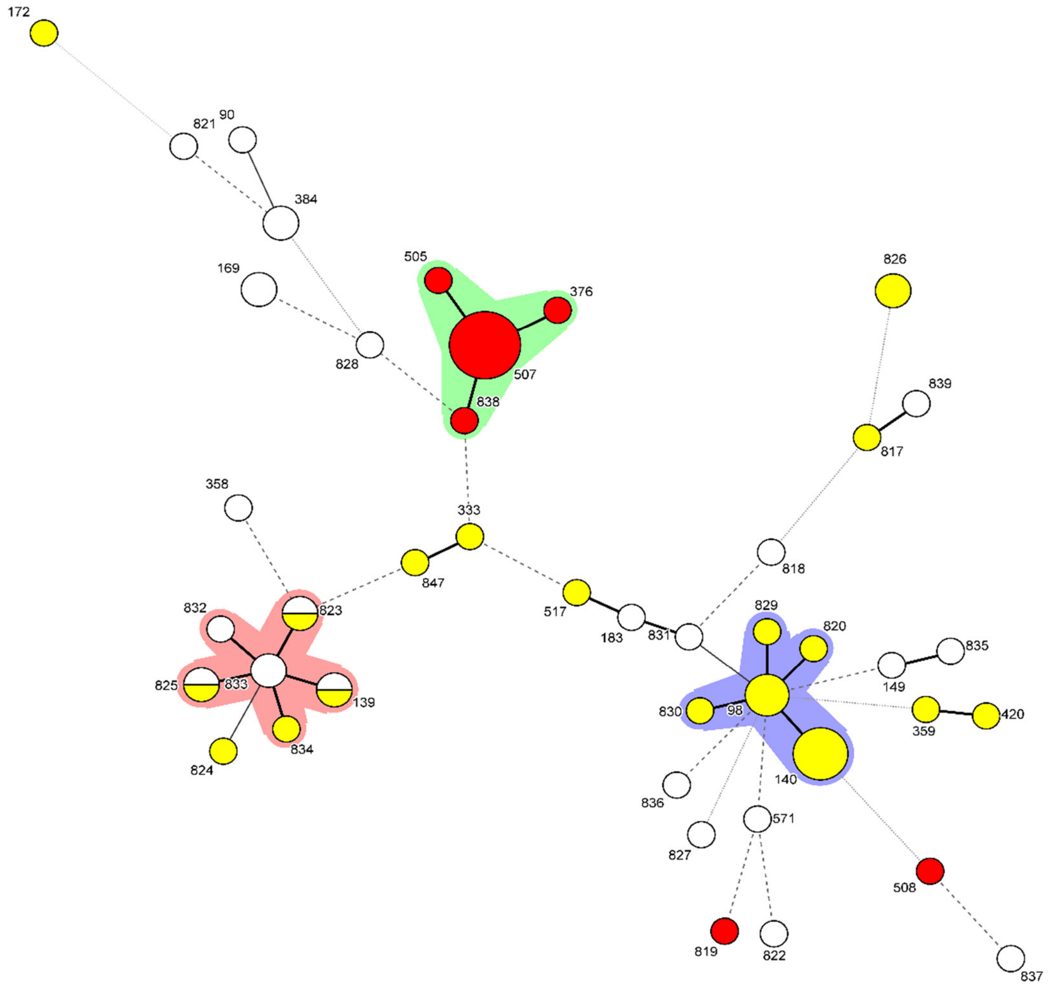

Supplementary Figure S2

Overview of the EUCAST microdilution ELISA read-outs of twelve isolates in clonal complex 3. The background OD of the ELISA reader has been subtracted. On the Y-axis, growth in individual wells is presented as mean growth percentages of twelve isolates compared to growth in the antifungal free growth control wells. Error bars indicate standard deviations. The dotted line indicates the 50% growth line (MIC values are read using a 50% inhibition endpoint).

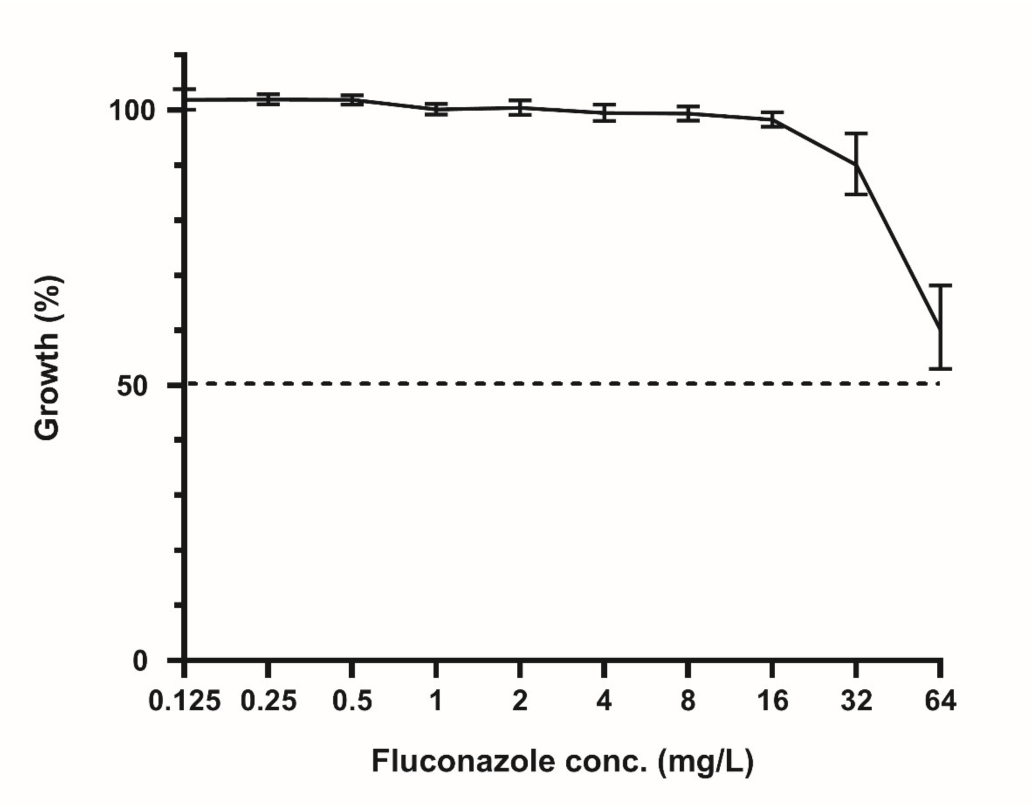

Supplementary Figure S3.

Scatter plot between geometric means of trailing percentages and *MDR1* expression levels. A solid line indicates the linear regression line and dotted lines indicate 95% confidence intervals.

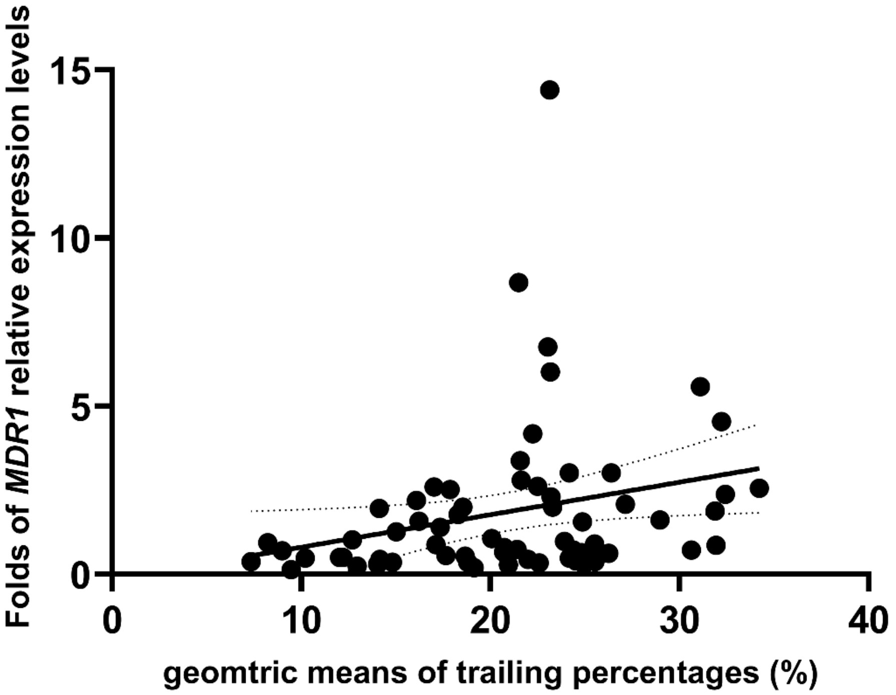

References

1. Vandeputte, P.; Larcher, G.; Berges, T.; Renier, G.; Chabasse, D.; Bouchara, J.P. Mechanisms of azole resistance in a clinical isolate of *Candida tropicalis*. *Antimicrob. Agents Chemother.* **2005**, *49*, 4608–4615, doi:10.1128/AAC.49.11.4608-4615.2005.
2. Forastiero, A.; Mesa-Arango, A.C.; Alastruey-Izquierdo, A.; Alcazar-Fuoli, L.; Bernal-Martinez, L.; Pelaez, T.; Lopez, J.F.; Grimalt, J.O.; Gomez-Lopez, A.; Cuesta, I., et al. *Candida tropicalis* antifungal cross-resistance is related to different azole target (Erg11p) modifications. *Antimicrob Agents Chemother.* **2013**, *57*, 4769-4781, doi:10.1128/AAC.00477-13.
3. Choi, M.J.; Won, E.J.; Shin, J.H.; Kim, S.H.; Lee, W.G.; Kim, M.N.; Lee, K.; Shin, M.G.; Suh, S.P.; Ryang, D.W.; et al. Resistance mechanisms and clinical features of fluconazole-nonsusceptible *Candida tropicalis* isolates compared with fluconazole-less-susceptible isolates. *Antimicrob. Agents. Chemother.* **2016**, *60*, 3653–3661, doi:10.1128/AAC.02652-15.
4. Jiang, C.; Ni, Q.; Dong, D.; Zhang, L.; Li, Z.; Tian, Y.; Peng, Y. The Role of *UPC2* Gene in Azole-Resistant *Candida tropicalis*. *Mycopathologia* **2016**, *181*, 833-838, doi:10.1007/s11046-016-0050-3.
